# MicroRNA profiling of cerebrospinal fluid from dogs with steroid responsive meningitis-arteritis and meningoencephalitis of unknown origin

**DOI:** 10.3389/fvets.2023.1144084

**Published:** 2023-05-05

**Authors:** Emilio Mármol-Sánchez, Pernille Lindholm Heidemann, Hanne Gredal, Susanna Cirera

**Affiliations:** ^1^Department of Molecular Biosciences, The Wenner-Gren Institute, Stockholm University, Stockholm, Sweden; ^2^Centre for Paleogenetics, Stockholm University, Stockholm, Sweden; ^3^Department of Veterinary Clinical Sciences, Faculty of Health and Medical Sciences, University of Copenhagen, Copenhagen, Denmark; ^4^Department of Veterinary and Animal Sciences, Faculty of Health and Medical Sciences, University of Copenhagen, Copenhagen, Denmark

**Keywords:** next generation sequencing, microRNA, dog, cerebrospinal fluid, steroid responsive meningitis-arteritis, meningoencephalitis of unknown origin

## Abstract

**Introduction:**

Non-infectious inflammatory diseases of the central nervous system in dogs, such as steroid responsive meningitis-arteritis (SRMA) and meningoencephalitis of unknown origin (MUO), represent a common clinical challenge that needs extensive and multimodal work-up to reach a presumptive diagnosis. Both diseases are presumably caused by dysregulations of the immune system, but further research is needed in order to understand the molecular mechanisms behind each disease and to optimize treatment.

**Methods:**

By next-generation sequencing and subsequent quantitative real-time PCR (qPCR) verification, we designed a prospective case–control pilot study to analyze the small RNA profiles of cerebrospinal fluid from dogs suffering from MUO (*N* = 5), dogs suffering from SRMA (*N* = 8), and healthy dogs (*N* = 5) presented for elective euthanasia used as the Control group.

**Results:**

Our results showed an overall enrichment in Y-RNA fragments across all samples, followed by microRNAs (miRNAs) and ribosomal RNAs as the major findings. Additional traces of short RNA reads mapped to long non-coding RNAs and protein-coding genes were also found. From the detected canine miRNAs, miR-21, miR-486, miR-148a, miR-99a, miR-191 and miR-92a were among the most abundant. Dogs with SRMA showed higher differences in miRNA abundance than dogs with MUO when compared to healthy dogs, and miR-142-3p was consistently detected as differentially upregulated in both diseases, although at a low concentration. Moreover, miR-405-5p and miR-503-5p showed different profiles between SRMA and MUO dogs. Subsequent qPCR analyses confirmed miR-142-5p, miR-191-5p and miR-92a-3p as significantly upregulated miRNAs in dogs with SRMA and/or MUO.

**Discussion:**

Cerebrospinal fluid is a challenging biological material to use for profiling miRNAs due to the low content of circulating RNAs. Despite this, we could confirm several miRNAs being differentially abundant when comparing healthy dogs and dogs with MUO and SRMA, respectively. The results of this study indicate a potential role of miRNAs in the underlying molecular mechanisms of these diseases and establish the basis for further studies.

## 1. Introduction

Meningoencephalitis of unknown origin (MUO) and steroid responsive meningitis-arteritis (SRMA) in dogs are both common non-infectious inflammatory diseases, affecting the meninges and/or the central nervous system (CNS) (1, 2). For both diseases, the underlying etiology is presumed to be an immune system dysregulation, but other than that, they are different disease entities, with MUO having a graver prognosis than SRMA (3). MUO is an umbrella term covering several encephalitides that can currently only be differentiated by histopathology (4). Clinical phenotypes for the different types of encephalitides overlap greatly from a clinical perspective, and studies examining MUO often do not differentiate dogs into pathological subgroups, hence MUO is used as a common term to cover very similar clinical phenotypes (1, 5, 6). MUO is usually diagnosed based on pathological changes on neuroimaging, i.e., magnetic resonance imaging (MRI) or computed tomography (CT), and cerebrospinal fluid (CSF) analysis. However, in some patients, both neuroimaging and CSF analysis are normal, making an ante-mortem diagnosis extremely challenging (5). For all subtypes, an early diagnosis and initiation of treatment is, however, of great importance, as early initiation of treatment has been shown to affect survival (7). Treatment is based on general immunosuppressive therapy, usually involving high dose corticosteroids and other immunomodulatory drugs in combination, often introducing a number of unwanted side effects, but as the specific underlying immunopathological mechanisms are largely unknown, targeted treatment toward these is not an option at present (6). A better understanding of the disease mechanisms is therefore warranted. SRMA, in contrast to MUO, is a systemic inflammatory disease affecting the meninges and meningeal arteries (8, 9), and usually responds well to treatment with corticosteroids (10). Dogs are commonly presented with cervical hyperesthesia, anorexia, reluctance to move and systemic inflammation (10). In the absence of specific serum biomarkers, the mainstay in diagnosing SRMA is analysis of CSF, which requires an invasive sampling procedure under general anesthesia (2, 11).

MicroRNAs (miRNAs) are a group of small non-coding RNAs that can be found in body fluids such as serum, CSF and saliva (12, 13). MiRNAs are known to play a post-transcriptional regulatory role in molecular pathways involved in multiple pathological processes, including non-infectious inflammatory CNS diseases. In humans, a relationship between the expression of some miRNAs in inflammation and neurodegenerative diseases such as Alzheimer’s and Parkinson’s diseases has been convincingly demonstrated (12, 14, 15). However, miRNA research in dogs is sparse so far. Previous studies have investigated selected miRNAs by probe-based hypothesis-driven methods, i.e., NanoString or microarray platforms, in a small number of diseases, including neurological and cardiac pathologies (16, 17). Research on miRNA abundance profiles in CSF in dogs with inflammatory neurological disease is also limited (16), and has proven to be challenging (18). Despite this, miRNAs remain of great interest, as they appear more stable in circulation than traditional biomarkers (19–22), qualifying their use and possible implementation in clinical research (18, 19). More importantly, the identification of changes in the abundance of certain circulating miRNAs in CSF from dogs suffering from MUO or SRMA has the potential to improve our knowledge of the mechanisms involved in each disease, hence ameliorating the treatment of affected patients.

This pilot study aimed to investigate CSF-miRNA abundance profiles by hypothesis-free methods, i.e., small RNA sequencing, and subsequent verification by qPCR, in order to shed light on the molecular mechanisms that characterize SRMA and MUO, and to potentially reveal novel targets for treatment.

## 2. Materials and methods

### 2.1. Animals

The study was performed as a prospective case–control study. CSF was collected from a total of 15 dogs allocated to three different groups: (i) MUO group (*N* = 5), (ii) SRMA group (*N* = 5), and iii) Control group (*N* = 5). Three additional dogs suffering from SRMA were included in the qPCR verification analyses.

For the MUO and SRMA groups, dogs with characteristic clinical and paraclinical findings as deemed by the responsible clinician at the Neurology service at the University Hospital for Companion Animals (Copenhagen, Denmark) were included. For the dogs with MUO, a pleocytosis in CSF dominated by mononuclear cells following general recommendations for a tentative diagnosis (23) was required for inclusion, supported by diagnostic imaging or necropsy when available. Besides the characteristic clinical presentation, dogs included in the SRMA group were required to have a pleocytosis in CSF, defined as >5 nucleated cells/μL dominated by neutrophilic granulocytes or monocytes as generally advised (23), as well as a systemic inflammatory response, defined as a C-reactive protein (CRP) above normal reference range (> 25 mg/L). In the SRMA and MUO groups, a positive response to treatment with immunosuppressive drugs was supportive of the diagnosis. Dogs were excluded from the two disease groups if they suffered from non-related inflammatory or systemic diseases, if a CSF tap was contraindicated and/or if they had been treated with nonsteroidal anti-inflammatory drugs or corticosteroids for >7 days prior to presentation. For the healthy Control group, dogs presented for elective euthanasia with no clinical neurological or systemic disease were included, provided they had a CSF analysis within standard reference values. This was defined as a total nucleated cell count (TNCC) ≤ 5 cells/μL, and a protein count <30 mg/dL for cerebellomedullary samples (23–25). In all groups, dogs were excluded if there were any signs of blood contamination on visual inspection of CSF or if the amount of red blood cells (RBC) compromised the cytological evaluation and the TNCC, defined as a maximum of 8,480 RBC/μL as previously reported for canine CSF (26). A comprehensive list of all dogs analyzed in the present study, as well as clinical results and follow-up from the analysis of their sampled CSF with CRP and TNCC measurements (in SRMA and MUO groups) is available at Table 1.

**Table 1:** Overview of signalment and relevant clinical data measured in cerebrospinal fluid (CSF).

	Age (months)	Gender	Weight (kg)	Breed	CRP	CSF: TNCC/μL	PR	CSF Cytology	Follow-up
Control (N = 5)									
C1	13	*M*	10–20	Medium mixed breed		1	0	Normal	
C2	50	*F*	41.2	Berner Sennen		2	0	Normal	
C3	110	*M*	40.7	Labrador Retriever		2	1	Normal	
C4	23	*M*	41.9	Medium mixed breed		3	0	Normal	
C5	15	*M*	>20	German Shepherd		1	0	Normal	
*Mean [range]*	*42.2* *[13–110]*					*1.8* *[1–3]*			
SRMA (N = 8)									
S1	13	*M*	31.5	Golden Retriever	264.7	29	1	NGd, mild/moderate mixed PL	RTT
S2	10	*F*	11.0	Whippet	111.9	1,660	1	NGd, moderate mixed PL	RTT
S3	27	*M*	29.6	Border Collie	251.5	133	0	NGd, marked mixed PL	RTT
S4	11	*F*	20.5	Golden Retriever	239.6	6,400	4	NGd, marked mixed PL	RTT
S5	25	*F*	11.8	Welsh Corgi Cardigan	120.6	192	1	NG PL	RTT
S6*	21	*F*	16.6	Small Münsterländer × Cocker Spaniel	71.0	18	0	NG PL	RTT
S7*	6	*M*	19.5	Border Collie × Golden Retriever	87.0	579	1	NG marked PL	RTT
S8*	8	*F*	32.2	Golden Retriever	158.9	101	0	NG PL	RTT
*Mean [range]*	*17.7* *[10–27]*		*20.9* *[11–31.5]*		*163.2* *[71–264.7]*	*1,139* *[18–6,400]*			
MUO (N = 5)									
M1	64	*M*	8.0	Chinese Crested Dog	2.8	1,175	2	LMCd, marked PL	Euthanized**
M2	68	*F*	3.2	Pražský Krysařík	21.2	911	1	MCd, marked mixed PL	RTT
M3	44	*F*	1.6	Yorkshire Terrier	1.8	299	3	LMCd, PL	Euthanized***
M4	39	*F*	2.7	Chihuahua	110.0	95	2	SMCd, mixed PL	Euthanized****
M5	15	*F*	4.7	Small Mixed Breed	3.4	11	0	Mixed MC and PL	RTT
*Mean [range]*	*46* *[15–68]*		*4.0* *[1.6–8]*		*8.0* *[1.8–21.2]*	*498* *[11–1,175]*			

MUO, meningoencephalitis of unknown origin; SRMA, steroid responsive meningitis-arteritis; M, male; F, female; CRP, C-reactive protein; TNCC, total nucleated cell count measured in CSF; PR, protein levels measured in CSF; NG, neutrophilic granulocytes; d, dominant; PL, pleocytosis; LMC, large mononuclear cells; MC, mononuclear cells; RTT, responded to treatment initially. For dogs included in the control group, CRP was not measured. For dogs with SRMA, initial dose of prednisolone ranged from 1.9-2.1 mg/kg/day. For dogs with MUO, initial dose of prednisolone ranged from 3.1-3.2 mg/kg/day. ^*^Additional dogs included in qPCR analyses but not used in initial small RNA sequencing. ^*^^*^Euthanized within 24 hours from diagnosis. Granulomatous meningoencephalitis was confirmed on histopathology. ^*^^*^^*^Euthanized 24 hours after diagnosis due to worsening of neurological signs - multifocal cerebral lesions seen on CT, rim-like contrast enhancement. Tentative diagnosis of necrotizing encephalitis. ^*^^*^^*^^*^Euthanized at diagnosis.

### 2.2. Sample collection and handling

Cerebrospinal fluid was collected *lege artis* from the cerebellomedullary cistern for all analyzed groups. In the two disease groups (MUO and SRMA), surplus CSF from the clinical work-up was included. In the event of death or euthanasia before CSF collection could be performed, CSF was collected within 30 min of euthanasia and included for routine analysis and miRNA investigations. In all healthy dogs, CSF was collected postmortem within 30 min of euthanasia. The time limit of 30 min from death was set to avoid postmortem changes to the CSF composition, as also applied in previous human studies (27).

Standard analysis of CSF for clinical verification was performed within 1 h of collection. The analysis included macroscopic inspection of color and turbidity, microscopic investigation of cell morphology, and analysis of protein level and nucleated cell content. The remaining CSF was centrifuged at 2000 g for 15 min at 4°C (Multifuge 1 S-R, Heraeus). Aliquots of the supernatant were then transferred to sterile cryotubes at a maximum volume of 200 μL/tube and stored at −80°C until RNA purification.

### 2.3. Purification of RNA and sequencing

RNA was purified from 200 μL of stored CSF samples using the miRNeasy Mini Kit (Qiagen, Hilden, Germany) following the manufacturer’s instructions. The quality and quantity of the purified RNA was evaluated using the Nanodrop 1,000 spectrophotometer (ThermoFisher Scientific). Six μL of RNA from each sample (*N* = 15) were shipped to the Center for Genomic Regulation (CRG), Genomic Service at Barcelona, Spain, for sequencing. Small RNA-seq libraries were prepared with the NEBNext® Small RNA Library Prep kit (New England Biolabs) and sequenced on an Illumina HiSeq 2,500 system to generate 50 bp single-end reads.

### 2.4. Small RNA-seq pre-processing, mapping, and quantification

Raw FASTQ files were processed for sequencing adapter trimming with the Cutadapt v3.2 software (28) and allowing a maximum of 10% error rate for adapter identification, as well as a minimum read length after trimming of at least 18 nucleotides (nt). Quality check filtering was performed with the fastp v0.12.4 software tool (29), allowing a qualified PHRED score per nucleotide ≥30. Reads fulfilling quality check requirements were then mapped against canine precursor miRNA sequences (pre-miRNAs, *N* = 502) belonging to the domestic dog genome assembly (CanFam3.1) according to the miRBase v22.1 database (30). Alignment was performed with the Bowtie tool v1.3.0 (31) using the following specifications tailored for small RNA sequences: i) no mismatches allowed, (ii) reporting the best alignment with high sensitivity within the stacked multimapping repertoire, and (iii) removing any reverse-complement match (*−v 0−k 1−y --best -no-rc*). Quantification of miRNA abundance was assessed independently for each 5p and 3p mature miRNA ends from each pre-miRNA detected.

In addition, we aimed to characterize the presence of RNA fragments belonging to transcripts derived from loci categories other than miRNAs (i.e., mRNAs, ribosomal RNAs, long non-coding RNAs or Y-RNAs). For this purpose, quality-filtered reads were mapped to the whole-genome assembly of the domestic dog (ROS_Cfam_1.0) by using the Bowtie v1.3. 0 aligner as implemented for miRNA-targeted mapping. We allowed up to a maximum of 1 mismatch with a mapping seed of 18 nt, equal to the minimum read length allowed (*−n 1−l 18−k 1−y --best*). Quantification was then performed with the featureCounts v2.0.3 tool (32) and focused on the exonic fraction of the successfully assigned reads. Gene annotations were retrieved from the latest Ensembl release available when performing the analyses (Cfam_1.0 v.105) (33).

### 2.5. Differential abundance

The significance of differences in miRNA abundance for Control vs. MUO dogs, Control vs. SRMA dogs, and MUO vs. SRMA dogs was assessed by using the edgeR tool (34). MiRNAs with extremely low abundance were tagged with the *filterByExpr* R function and removed from differential abundance analyses. Abundance-filtered raw counts were then normalized for library depth with the trimmed mean of M-values normalization (TMM) method (35). Statistical significance of mean abundance differences was tested with a quasi-likelihood F-test (34). Multiple hypothesis testing correction was implemented with the false discovery rate (FDR) method (36). Significant differential abundance was set at an absolute fold-change (FC) value >2, i.e., |log_2_FC| > 1 in the log_2_ scale, and FDR corrected *p*-value (*q*-value) < 0.05. Relevant miRNAs showing significant differential abundance and, whenever possible, high abundance, were selected for qPCR assessment. A comprehensive pipeline of bioinformatics approaches for RNA pre-processing, mapping, quantification, and differential abundance analyses is shown in Supplementary Figure S1.

### 2.6. Validation of candidate miRNAs using qPCR

Three additional dogs were recruited for the SRMA group after the sequencing part of the project was finished and were included for qPCR verification. RNA extraction for these three additional samples was done as described above. No additional RNA was available for dog M2 from the MUO group after sequencing, hence it was excluded from qPCR analyses. Two μL of RNA from each sample were used for cDNA synthesis according to methods reported previously (37). For each RNA sample, two replicates of cDNA were done when enough RNA was available (for dogs M1, M3 and M4 from the MUO group, as well as for dogs S1, S2, S4 and S5 from the SRMA group, only 1 replicate could be done). Negative technical controls (NTC) without adding cDNA to the reaction mix, as well as control samples with cDNA synthesized but not including poly(A) polymerase (noPAP) were also included. The cDNAs were finally diluted eight times and used for qPCR procedure.

A panel of 18 miRNAs were included in the verification phase. Specific forward and reverse primers were designed for each miRNA selected according to the miRprimer software (38) following protocols described previously (39). Primer sequences are shown in Supplementary Table S1. qPCR was done in a Mx 3005P machine (Agilent) using QuantiFast SYBR Green Master Mix (Qiagen) with the following cycling conditions: Five minutes at 95°C, 40 cycles of 10 s at 95°C and 30 s at 60°C, followed by melting curve analysis to ensure specific amplification. Data was manually curated and assays with no Cq (quantification cycle) values or values >33 cycles were excluded. NTC and nonPAP control samples were visually inspected and considered valid if showing no amplification or amplification with Cq at least >5 cycles away from CSF samples. Data pre-processing was done using GenEx Pro v.6 software (MultiID, Sweden). Briefly, data was normalized to the most stable miRNA (cfa-let-7a) according to the NormFinder algorithm (40). Technical repeats were then averaged. Subsequently, relative quantities were calculated by scaling to the lowest abundant sample for each assay. Finally, data was log_2_ transformed to run statistical analyses. A t-test with Welch correction was performed for the following comparisons: Control vs. MUO, Control vs. SRMA and MUO vs. SRMA. Multiple testing correction was implemented by using the FDR approach (36). MiRNAs with FC > 2 and *q*-value <0.05 were considered as significantly deregulated by qPCR technique. Raw data from qPCR and subsequent intermediate processing steps are shown in Supplementary Table S2.

### 2.7. Tissue clustering

A miRNA tissue atlas from the domestic dog was used to infer whether the miRNA abundance profiles obtained from CSF in our study resembled that from any of the available canine reference tissues and their homogeneity. In this way, an overall equivalent miRNA abundance among the CSF samples would result in an adjacent clustering of CSF miRNA profiles among each other. Moreover, similar miRNA abundance patterns to other tissues would be reflected in their proximity to the corresponding samples from the dog miRNA atlas, thus indicating the putative tissue origin of the miRNAs present in CSF. Small RNA-seq samples for building the dog tissue atlas were retrieved (41, 42) and mapped against the reference domestic dog miRNA annotation according to miRGeneDB 2.1 (43). To allow full equivalence, small RNA-seq data from CSF obtained in this study was also mapped to dog primary miRNAs (pri-miRNAs) available at miRGeneDB 2.1 (43). The miRNA quantification of each considered reference dog tissue (hypothalamus, cerebellum, brain, sciatic nerve, bone marrow, pancreas, lung, liver kidney, plasma, heart, and skeletal muscle), as well as of CSF samples, was normalized within tissue by transforming individual counts to counts-per-million (CPM) values with respect to the total reads successfully mapped to the whole canine genome (ROS_Cfam_1.0). The *umap* function from the umap v0.2.7.0 R package^1^ was then implemented to determine a dimensionality reduction of the reference dog tissue atlas (*n_neighbors = 15, metric = “pearson,” spread = 15, random_state = 123*) using the uniform manifold approximation and projection (UMAP) algorithm (44). Finally, the CSF miRNA profiles were projected onto the previously learned UMAP embedding.

### 2.8. MiRNA target prediction

The TargetScan v8.0 (45) with *H. sapiens* reference database was employed to predict putative highly conserved mRNA targets for three of the miRNAs (cfa-miR-142-5p, cfa-miR-191-5p and cfa-miR-92a-3p) detected as significantly upregulated in the CSF pathological groups (MUO and/or SRMA) by both small RNA-seq and qPCR techniques. Since the seed region (2^nd^ to 8^th^ nt from the 5′ end of the mature miRNA sequence) of the selected miRNAs is highly conserved among human, domestic dog and many other mammals, we prioritized highly conserved mRNA targets as predicted by the TargetScan algorithm. In this way, we sorted the predicted mRNA targets for each of the three miRNAs by their aggregate P_CT_ score, which represents an approximation to the probability of a given miRNA-mRNA interaction to have highly conserved functionality across mammals (46). Only predicted mRNA targets with P_CT_ score > 0.4 were used for further analyses.

### 2.9. Pathway enrichment

The putative mRNA targets predicted for cfa-miR-142-5p, cfa-miR-191-5p and cfa-miR-92a-3p miRNAs according to TargetScan v8.0 (45) were then subjected to pathway enrichment analysis using the ClueGO v.2.5.7 plug-in application (47) within the Cytoscape v.3.6 (48) software. We implemented the KEGG pathway human database (v.2020) as reference, jointly with a right-sided hypergeometric test for gene enrichment calculation. Pathways were considered as significantly enriched if they were represented by at least five predicted targeted mRNA genes by the set of selected miRNAs, and a *q*-value <0.05 after multiple testing correction with the false discovery rate (FDR) approach (36).

## 3. Results

### 3.1. Dog population and clinical manifestation

The current pilot study included three different groups of dogs (MUO, *N* = 5; SRMA, *N* = 5 for sequencing and *N* = 8 for subsequent qPCR analysis; and healthy Control dogs, *N* = 5). A variety of breeds and weights were represented in the included cohort of dogs (Table 1), with dogs in the MUO group having a lower average weight compared to dogs suffering from SRMA, as well as healthy Control dogs. This was not a surprising finding, as MUO is known to affect small sized breeds in particular (5). The CRP levels for SRMA dogs were 20-fold higher than in MUO dogs (Supplementary Figure S2), while TNCC was highly variable in SRMA dogs, with S4 having ~14-fold higher TNCC than the average of the remaining SRMA dogs (Table 1). When disregarding S4 sample from the SRMA group, considered as an extreme outlier for the TNCC phenotype, SRMA dogs had a median TNCC value of 133 TNCC/μL, while MUO dogs showed a median of 299 TNCC/μL (Supplementary Figure S2). Moreover, SRMA dogs had an increased standard deviation for TNCC (*s* = 592.46) compared to MUO dogs (*s* = 516.739, Supplementary Figure S2).

### 3.2. Small RNA-seq analysis

An average of 15.7 million reads were successfully generated for each CSF sample after sequencing, except for one of the healthy Control samples (C3), which failed during library preparation and was hence excluded from further analyses based on small RNA-seq data (MUO, *N* = 5; SRMA, *N* = 5; and Control, *N* = 4). From the total number of raw RNA reads produced per sample, an average of 90.43% were successfully trimmed and passed the minimum length filtering criterion (at least 18 nt after adapter trimming), and 96.85% of these successfully passed quality-check filtering.

**Figure 1:**
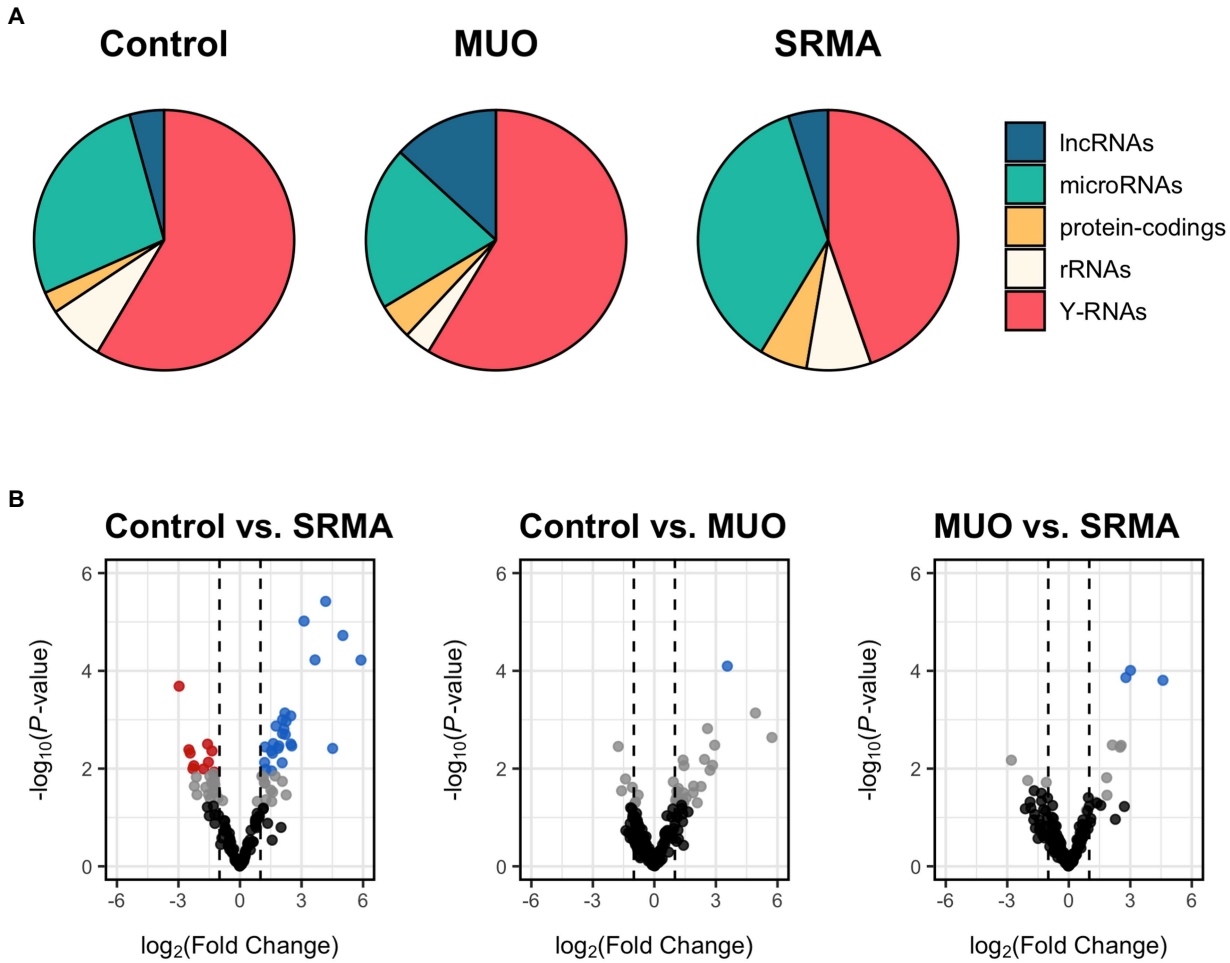
**(A)** Pie charts depicting the proportion of mapped reads from small RNA-seq data to different types of annotated loci in healthy dogs (Control), dogs with meningoencephalitis of unknown origin (MUO) and dogs with steroid responsive meningitis-arteritis (SRMA). Among the most abundant loci represented are long non-coding RNAs (lncRNAs), miRNAs, protein-coding genes, ribosomal RNAs (rRNAs) and Y-RNAs. **(B)** Volcano plots depicting miRNAs significantly upregulated (log_2_FC > 1; *q*-value <0.05, in blue) or downregulated (log_2_FC < −1; *q*-value <0.05, in red) for dogs with MUO or SRMA with respect to healthy Control dogs according to small RNA-seq data. For the MUO vs. SRMA contrast, upregulated miRNAs (in blue) were considered as those showing increased abundance in the SRMA group with respect to the MUO group, and vice versa. In grey are miRNAs showing nominal *p*-value <0.05. In black are miRNAs showing nominal *p*-value >0.05.

Genome-wide mapping to the domestic dog assembly (ROS_Canfam_1.0) produced a variable number of successful alignments, ranging between 28.35 and 82.29%. From these, between 3.9 and 46.5% of the aligned reads mapped to annotated genes in the dog assembly. Roughly half of the reads mapped to annotated loci belonged to Y-RNAs, followed by miRNAs, ribosomal RNAs (rRNAs), long non-coding RNAs (lncRNAs) and traces of mRNA transcripts from protein-coding genes (Figure 1A). A comprehensive list and abundance profiles of all quantified loci genome-wide in each defined CSF group is shown in Supplementary Table S3.

For reads mapped to dog miRNAs according to the miRBase reference (*N* = 502), between 1.29 million and 29 thousand reads were successfully assigned. Principal component analysis (PCA) based on miRNA abundance for each of the 14 CSF samples included initially revealed that sample S4 (belonging to the SRMA pathological group) showed a clear separation over principal component 1 (92.45% of explained variance, Supplementary Figure S3A), while the other samples clustered more closely. Upon analyzing the overall clustering of the PCA without including sample S4, which was previously considered as an extreme outlier for TNCC (Supplementary Figure S3B), we decided to remove this sample for further analyses, retaining 4 Control, 4 SRMA and 5 MUO CSF samples for differential abundance assessment.

### 3.3. miRNA differential abundance

Differential abundance analyses were performed by using CSF from healthy Control samples as reference; hence, any obtained upregulation would imply an overexpression of the given miRNA in either SRMA or MUO group with respect to the Control group, and vice versa. Control samples (*N* = 4) were compared separately with SRMA (*N* = 4) and MUO samples (*N* = 5). Additionally, SRMA and MUO samples were compared independently to assess the presence of discriminative differentially abundant miRNAs between both diseases. In this way, MUO samples (*N* = 5) were considered as reference, meaning that any given miRNA upregulation would imply an overexpression of the miRNA in the SRMA group with respect to the MUO group, and vice versa.

Only one very lowly abundant miRNA (cfa-miR-142-3p) was detected as significantly upregulated in MUO dogs compared to healthy Control dogs (|log_2_FC| > 1, *q*-value <0.05; Supplementary Table S4, in grey, and Figure 1B, in blue), while a total of 36 miRNAs were significantly differentially abundant in Control vs. SRMA (26 upregulated and 10 downregulated in SRMA dogs compared to healthy dogs, Supplementary Table S4, in grey, and Figure 1B). Of these, 19 (52.77%) showed average abundance levels above 100 read counts. Regarding the contrast between MUO and SRMA samples, three lowly abundant miRNAs were detected as differentially upregulated in SRMA dogs (cfa-miR-450b-5p, cfa-miR-450a-5p and cfa-miR-503-5p, Supplementary Table S4, in grey, and Figure 1B, in blue). A complete list of differential abundance analyses for all analyzed miRNAs by small RNA-seq is available in Supplementary Table S4.

**Figure 2:**
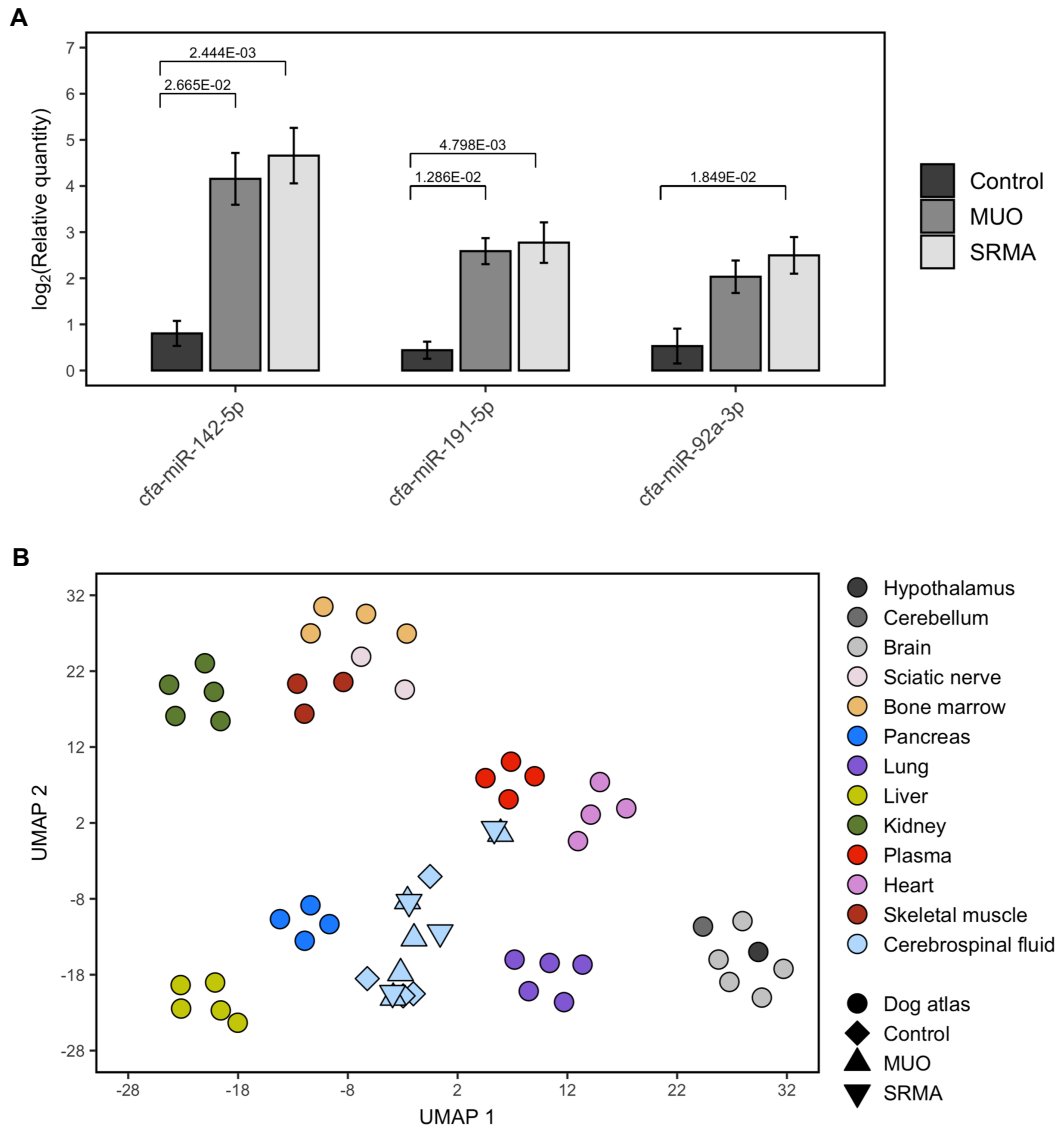
**(A)** Barplots depicting qPCR log_2_ transformed relative quantities and their significance levels for cfa-miR-142-5p, cfa-miR-191-5p and cfa-miR-92a-3p miRNAs measured in cerebrospinal fluid (CSF) from healthy dogs (Control), dogs with meningoencephalitis of unknown origin (MUO) and dogs with steroid responsive meningitis-arteritis (SRMA). **(B)** UMAP plot depicting sample clustering of a collection of tissues from a canine miRNA expression atlas including miRNA profiles of hypothalamus, cerebellum, brain, sciatic nerve, bone marrow, pancreas, lung, liver kidney, plasma, heart, and skeletal muscle. The sample clustering for miRNA profiles from CSF samples based on small RNA-seq data of healthy dogs (Control, *N* = 4), dogs with MUO (*N* = 5) and dogs with SRMA (*N* = 4) was then predicted using the learned UMAP embedding using the initial dog miRNA expression atlas.

After qPCR verification, two significantly upregulated miRNAs (cfa-miR-191-5p and cfa-miR-142-5p) were detected in MUO dogs with respect to Control healthy dogs (|FC| > 2, *q*-value <0.05, Table 2 and Figure 2A). Their abundance change agreed with that obtained in small RNA-seq analyses, although using sequencing technique they were only detected as significant at nominal *p*-value <0.05 (Supplementary Table S4). Interestingly, three miRNAs (cfa-miR-191-5p, cfa-miR-142-5p and cfa-miR-92a-3p) were also detected as differentially upregulated by qPCR when comparing SRMA dogs with Control healthy dogs (Table 2; Figure 2A), from which two of them (cfa-miR-191-5p and cfa-miR-142-5p) were also significantly upregulated in the MUO group by qPCR, and all three were significantly differentially abundant according to small RNA-seq data in the Control vs. SRMA contrast (Supplementary Table S4, in grey). No significant differences were found when comparing MUO with SRMA dogs by qPCR. A complete list of differential abundance analyses for all analyzed miRNAs by qPCR is available in Supplementary Table S5.

**Table 2:** Differentially abundant miRNAs (|FC| > 2; *q*-value <0.05) detected by qPCR when comparing Control vs. MUO dogs and Control vs. SRMA dogs.

miRNA	FC	*p*-value	*q*-value
Control vs. MUO			
miR-191-5p	5.881	1.071E-03	1.286E-02
miR-142-5p	5.168	4.442E-03	2.665E-02
Control vs. SRMA			
miR-142-5p	5.794	2.037E-04	2.444E-03
miR-191-5p	6.301	7.997E-04	4.798E-03
miR-92a-3p	4.710	4.622E-03	1.849E-02

FC, Fold change of the log_2_ Relative quantity (RQ) values of MUO or SRMA group with respect to RQ values of the Control group; *q*-value = *p*-value corrected for multiple testing with the false discovery rate (FDR) approach. Any observed miRNA upregulation (positive FC) implies an overexpression of the miRNA in the SRMA or MUO group with respect to the Control group, and vice versa.

### 3.4. Pathway enrichment analysis

Among the three queried miRNAs that showed significant abundance difference between healthy and disease states in CSF by small RNA-seq and qPCR (cfa-miR-142-5p, cfa-miR-191-5p and cfa-miR-92a-3p), two of them (cfa-miR-142-5p and cfa-miR-92a-3p) gathered significantly enriched pathways after mRNA target prediction (Supplementary Table S6). A good proportion of the pathways highlighted as significant were related to immune response, such as TGF-β signaling pathway (KEGG:04350), MAPK signaling pathway (KEGG:04010), bacterial infection (KEGG:05135, KEGG:05100), leukocyte migration (KEGG:04670), FoxO signaling pathway (KEGG:04068), cAMP signaling pathway (KEGG:04024) or Hippo signaling pathway (KEGG:04392). All predicted highly conserved and putatively targeted mRNAs used for enrichment analyses are shown in Supplementary Tables S7-S9.

### 3.5. Tissue clustering

We analyzed the putative tissue/cell origin of the miRNAs quantified in the dog CSF samples by using a reference dog miRNA atlas with a total of 12 different tissues (i.e., hypothalamus, cerebellum, brain, sciatic nerve, bone marrow, pancreas, lung, liver, kidney, plasma, heart and skeletal muscle) and our small RNA sequencing data. After tissue clustering based on miRNA expression profiles, the projection of CSF miRNA profiles using the UMAP algorithm resulted in an overall positioning of CSF samples as an independent cluster compared to other tissues, although two of them (M5 and S1, belonging to MUO and SRMA group, respectively), were located adjacent to dog plasma (Figure 2B).

## 4. Discussion

In the present study, we have investigated the profiles of cell-free small non-coding RNAs in canine CSF, with a specific focus on miRNAs, and included three experimental groups of dogs: a Control group consisting of healthy dogs, and two disease groups with dogs suffering from either MUO or SRMA. We first used small RNA sequencing on CSF samples, which provides higher sensitivity to quantify low abundant transcripts than qPCR, followed by subsequent verification of the best discriminating and abundant candidates by qPCR. Our goal in this pilot study was to shed light on the underlying immune mechanisms of SRMA and MUO.

To our knowledge, only a few studies have profiled miRNAs in CSF of dogs with MUO (49) or with MUO and SRMA (18). By using solely qPCR technique, Gaitero and coworkers identified higher levels of expression of miR-21 and miR-181c in the CSF from MUO dogs compared to dogs with non-inflammatory neurological diseases (49). Furthermore, they found a positive correlation between CSF cellularity and CSF expression of miR-21 in the MUO group (49). An additional study profiled miRNAs by qPCR using CSF of dogs with neoplastic, inflammatory, and degenerative disorders affecting the CNS (50). The authors found a significantly higher abundance of miR-10b-5p in the neoplastic group compared to other groups, but no correlation between miRNA expression and CSF cellularity nor CSF protein content. Moreover, in a previous report of our group using serum and CSF samples, we investigated the abundance of several miRNAs in dogs with MUO (*N* = 7) and SRMA (*N* = 6) using qPCR, where we managed to confirm the presence of several miRNAs, including miR-21, but struggled to reproduce consistent results in CSF (18). In the current study, we were only able to detect cfa-miR-142-3p as significantly differentially abundant in MUO dogs compared to healthy Control dogs using small RNA sequencing data. Moreover, qPCR verification highlighted cfa-miR-142-5p and cfa-miR-191-5p as significantly upregulated in MUO dogs, but both were only detected as significant at the nominal *p*-value with small RNA-seq data. However, cfa-miR-191-5p was detected as highly abundant according to small RNA sequencing, nearly 6-fold more abundant than cfa-miR-142-5p in MUO and SRMA dogs and might be a more interesting miRNA to be considered. For the dogs in the SRMA group, we were able to identify statistically significant levels of a total of 36 miRNAs using small RNA sequencing of CSF (see Results), and 3 out of the 12 miRNAs (cfa-miR-142-5p, cfa-miR-191-5p and cfa-miR-92a-3p) analyzed by qPCR were also significantly upregulated in SRMA dogs when compared to the Control group.

Focusing on the three differentially abundant miRNAs found in this study (cfa-miR-142-5p, cfa-miR-191-5p and cfa-miR-92a-3p), miR-142-5p has emerged as one of the most critical miRNAs during development, homeostasis, and disease, with important functions in infection and inflammation (51). Also, significant changes in its abundance have been found in plasma of Alzheimer’s disease compared to healthy subjects (52, 53). Moreover, the nuclear factor erythroid 2-like 2 (*NRF2*) is an experimentally validated target of miR-142-5p, and it is a relevant regulator involved in the response to injury and inflammation (53). Talebi and coworkers (54) showed that the miR-142 mature miRNA transcripts (−3p and -5p) in brain tissue of a mouse model for multiple sclerosis could target mRNAs encoding proteins involved in cytokine signaling and T cell differentiation, and confirmed that *SOCS1* mRNA, a negative regulator of cytokine signaling, is also a direct target of miR-142a-5p.

Regarding miR-191-5p, its expression has been shown to be stable in human CSF from the first year of life (55), it is abundantly expressed in the brain, and it has been associated with cell differentiation, proliferation, and metastasis (56). It has been found upregulated in plasma of patients with prolonged disorders of consciousness after severe brain damage (57). Furthermore, it has been proposed as a promising serum biomarker in Alzheimer’s disease (58).

Finally, miR-92a-3p belongs to the miR-17-92 cluster and it has been identified as a biomarker for many human cancers, acting as a tumor-promoting regulator through the PI3K/AKT/mTOR signaling pathway (59). Additionally, it has been found downregulated in plasma from Alzheimer’s disease patients (60).

Pathway enrichment analyses of putative predicted mRNA targets for cfa-miR-142-5p and cfa-miR-92a-3p highlighted several signaling pathways closely associated with immune response regulation, such as TGF-β (61), MAPK (62), FoxO (63), cAMP (64) or Hippo (65), as well as pathways related to bacterial infection and leukocyte migration. These results might indicate a link between an active immune response elicited by different types of leukocytes in the CSF. This might be explained by migration of leukocytes from the blood stream to CSF due to inflammation or, alternatively, blood contamination related to CSF collection. Nevertheless, blood contamination, if present, seems to have not affected the results of the present study as only two samples belonging to MUO and SRMA disease groups (M5 and S1) clustered adjacent to blood plasma samples. Overall, miRNA abundance in CSF samples did not resemble any of the tissues present in the miRNA expression atlas employed according to tissue clustering. Since no reference immune cell populations were present in our clustering analyses, it is difficult to infer a cellular origin of the profiled miRNAs in CSF. However, the close relationship with immune response predicted for the mRNA targets of cfa-miR-142-5p and cfa-miR-92a-3p may further confirm the hypothesis of both MUO and SRMA as immune-mediated diseases.

When we attempted to detect specific miRNAs differentiating MUO from SRMA dogs in the sequencing data, only 3 miRNAs were highlighted (cfa-miR-450a-5p, cfa-miR-450b-5p and cfa-miR-503-5p), but none of them were successfully profiled by qPCR, probably due to their very low abundance as detected by small RNA-seq. Interestingly, all these three miRNAs are reported to be involved in cancer development (66–68).

MiRNA studies in CSF have not previously been performed in dogs with SRMA apart from a few cases included in a population of various CNS diseases (50), and a previous work from our team where we were unsuccessful in profiling CSF by qPCR alone (18). Hence, similar canine studies for comparison are not available. In contrast to MUO, SRMA is characterized by systemic inflammatory changes, and several of the conventional biomarkers of systemic inflammatory conditions, e.g., CRP, fibrinogen, serum amyloid A, and IgA, are also elevated in dogs with SRMA (69–72). Moreover, SRMA is a well-characterized disease with well-described pathological findings (49). In this study, the SRMA group did not meet the same challenges as in the MUO group, since the study population consisted of a more homogeneous group of dogs, all of which fitted the characteristic description for the disease, both clinically and para-clinically. This might explain the higher significance in terms of abundance change detected in dogs suffering from SRMA when compared to the Control group, as opposed to the limited results obtained for dogs suffering from MUO. All dogs had a signalment resembling what has previously been reported regarding age, size, and breed (72, 73), and all showed cervical pain, as well as signs of systemic inflammation, including an increase in CRP above normal reference range (74). Furthermore, CSF analysis showed a pleocytosis dominated by neutrophilic granulocytes in all included dogs, as is classically seen in SRMA (23).

Several clinical challenges arose when recruiting healthy dogs, dogs suffering from SRMA, and especially when recruiting dogs suffering from MUO for CSF collection:

(i) The CSF of the Control group was collected upon euthanasia for ethical reasons, as opposed to most samples in the MUO and SRMA groups where CSF was taken as part of the diagnostic work-up. This might have biased the findings of the present study. Nevertheless, all CSF samples were collected within 30 min of euthanasia, in order to avoid postmortem changes to the CSF composition as suggested in human studies (27). These studies indicated, that if death is rapid, the endothelial lining of the cerebral blood vessels remains intact for hours, meaning that the total nucleated cell count would remain at the same level as at the time of death. No significant rise in protein count was observed within the first 24 h from death either. This, together with the high stability demonstrated by circulating miRNAs, provide robustness to the reliability of results reported in the current study.

(ii) The clinical diagnosis of MUO covers a rather heterogeneous group of non-infectious encephalitides, that, due to similar clinical presentation, can only be differentiated using histopathology. The main subtypes include necrotizing meningoencephalitis (NME), necrotizing leukoencephalitis (NLE) and granulomatous meningoencephalitis (GME), each characterized by specific histopathological findings, which presumably mirror specific underlying immune mechanisms (75). Ideally, future miRNA studies should be performed on dogs with a histopathologically confirmed MUO subtype (NME, NLE, GME). This would possibly offer a study of well-defined subgroups of MUO with characteristic changes in miRNA profiling. However, this approach would introduce several challenges, and a selection bias toward the more severe cases, as histopathology is usually only performed postmortem. Dogs that respond to treatment and survive would therefore not be included in a study where a histopathological diagnosis is an inclusion criterion.

(iii) The biological material used in the present study was CSF, since CSF is known to be the vehicle for intracerebral transport of biologically active substances (24), as well as having a low cell composition in both healthy and pathological states. The low cell composition leads to lesser “transcriptional noise” in sequencing that might be present in bodily fluids with a higher cell and protein content, such as serum. On the other hand, the levels of some relevant miRNAs in CSF may be too low to be confirmed by qPCR as it happened in the present study. Moreover, CSF biomarkers are not ideal for testing in a clinical setting, as CSF collection is an invasive procedure that is only performed under generalized anesthesia. For dogs suffering from SRMA, a tentative diagnosis is usually based on results of CSF analysis when seen in the context of clinical signs, signalment and other paraclinical results, i.e., signs of systemic inflammation (71). A biomarker that can only be identified in CSF is therefore less appealing. For miRNAs to be used as a biomarker in the clinical setting, identification of specific miRNAs in other easily accessible bodily fluids, such as serum or urine, would therefore be preferable. If that was possible, miRNA-profiling could have the potential to replace CSF analysis in dogs where SRMA is the most likely diagnosis.

(iv) Sampling of the CSF in small dogs (as representing the typical case of MUO) can be challenging because only a small amount of CSF can be safely collected. After performing small RNA sequencing, very little amounts of RNA isolated from CSF were left for qPCR verification. Sampling a bigger volume of CSF might be challenging, as the traditional recommendations for CSF collection involve a maximum of 1 mL/5 kg bodyweight (25). For four out of the five dogs included in the MUO group, the weight was below 5 kg. Unfortunately, a standard CSF analysis takes approximately 250 μL, and a minimum of 200 μL are needed for ensuring enough RNA concentration to perform RNA sequencing, leaving very little research material, if any, in dogs smaller than 5 kg.

The RNA isolation method employed in the present study allows purification of cell-free circulating small RNA biotypes including those inside extracellular vesicles (EV) and those attached to protein complexes (Ago2 or HDL). MiRNAs and other small ncRNAs are detected in body fluids at different concentrations depending on the investigated fluid and the experimental group (healthy versus disease). Many of these circulating small RNA biotypes other than miRNAs seem to be derived from larger RNAs as a result of processing events or degradation. In this study, a high percentage of sequencing reads derived from CSF-RNA mapped to Y-RNAs in all 3 experimental groups (with percentages ranging from 44 to 58%); followed by miRNAs (20–36%) with the higher representation in the SRMA group; lncRNAs (4–13%); rRNAs (3–8%) and protein-coding genes (2.6–6%). Of the various RNA biotypes found in circulation, the main focus of this study has been on miRNAs due to their known role in gene-regulation. Nevertheless, a very abundant class of RNAs associated with EV are the Y-RNAs; in fact, in the present study, they were the most abundant class of small non-coding RNAs in CSF. Y-RNAs have been previously found in all biofluids, including CSF (76, 77), which has generated interest in their potential use as disease biomarkers. Intact Y-RNAs have been associated with DNA replication and RNA stability (78). Accumulating evidence suggests that extracellular Y-RNAs present in biofluids may have immune-related functions, and changes in their circulating concentration have been associated with disease (79). Nevertheless, the exact role of fragments derived from Y-RNA genes found in CSF in the present study and their association to MUO and SRMA pathologies, if any, is not clear and further studies are warranted.

In summary, our results should be interpreted as a pilot study setting the bases for larger research projects. We hence recommend for future studies that: (i) they include a larger group of patients, and (ii) they include both serum and CSF for the examination of miRNAs identified as relevant in the SRMA group. If future studies successfully identify miRNAs related to SRMA in serum, there is a potential to develop a less invasive method for diagnosing SRMA. Moreover, miRNA profiling of both the aqueous phase of serum and CSF, as well as their nucleated cellular complement, could provide a more comprehensive view of the origin and biological function of the circulating miRNAs highlighted. In future studies regarding SRMA, we suggest to include two control groups. Control groups ideally include one group of healthy dogs, but also one group of dogs showing symptoms resembling SRMA, i.e., the clinical differential diagnoses, such as dogs suffering from, for instance, immune-mediated polyarthritis or infectious meningitis. This would further qualify miRNAs as potential biomarkers for SRMA in the clinical setting, as this is dependent on their ability to differentiate dogs with SRMA from dogs presenting with similar clinical symptoms, but another underlying pathology. For the MUO group, future studies would potentially benefit from subdivisions based on histopathology of patients (GME, NME and NLE), to identify relevant miRNAs in this disease group. Subdivision could potentially allow a better understanding of the differences between MUO subtypes and help optimize treatment for each.

## 5. Conclusion

In this pilot study, we have characterized the miRNA composition of the cerebrospinal fluid (CSF) in healthy dogs, dogs with steroid responsive meningitis-arteritis (SRMA) and dogs with meningoencephalitis of unknown origin (MUO). Our analyses based on small RNA-seq and qPCR verified three miRNAs (miR-142-5p, miR-191-5p and miR-92a-3p) present in canine CSF at differential abundance, which would be able to discriminate healthy dogs from dogs with MUO or SRMA. The detected differentially abundant miRNAs were predicted to have biological functions related to the immune response against the ongoing inflammation in dogs suffering from SRMA or MUO. The relatively few miRNAs identified to have discriminatory potential to differentiate healthy from pathological states in CSF might reflect the complex and poorly defined MUO pathology and the diverse subclasses included within this diagnosis, as well as the challenges of accurately profiling circulating RNAs in CSF. Future studies may benefit from a comprehensive differentiation of pathological subtypes within MUO, and the incorporation of additional controls and sequencing information coming from other biofluids, such as serum. Moreover, adding transcriptomes from the cellular fraction present in CSF and serum will help disentangle and differentiate the immune response activated in domestic dogs affected by MUO and SRMA.

## Data availability statement

The datasets presented in this study can be found in online repositories. The names of the repository/repositories and accession number(s) can be found at: Sequence Read Archive (SRA), accession number PRJNA921866.

## Ethics statement

The study was approved by the local ethical committee (permission number 2022–14) at the Department of Veterinary Clinical Sciences, University of Copenhagen. Written informed consent was obtained from the owners for the participation of their animals in this study.

## Author contributions

EM-S did the bioinformatic analysis on RNA-seq and qPCR data. PH recruited the dogs, performed the clinical work-up, and characterization as well as collection of samples. HG assisted in the clinical work-up and sample collection, secured funding, and helped directing the project. SC did the RNA isolation, qPCR analyses, secured funding, and coordinated the whole project. All authors contributed to the article and approved the submitted version.

## Funding

This study was funded by AGRIA Dyreforsikring Fund (Project nr: N2018-0004), Sweden.

## Conflict of interest

The authors declare that the research was conducted in the absence of any commercial or financial relationships that could be construed as a potential conflict of interest.

## Publisher’s note

All claims expressed in this article are solely those of the authors and do not necessarily represent those of their affiliated organizations, or those of the publisher, the editors and the reviewers. Any product that may be evaluated in this article, or claim that may be made by its manufacturer, is not guaranteed or endorsed by the publisher.

## Footnotes

^1^ https://github.com/tkonopka/umap
