# Supplementary material for: MicroRNA profiling of cerebrospinal fluid from dogs with steroid responsive meningitis-arteritis and meningoencephalitis of unknown origin
Source: Front Vet Sci. 2023 May 5;10:1144084. doi: 10.3389/fvets.2023.1144084 (PMC10196361; doi:10.3389/fvets.2023.1144084)
Supplement: Supplementary file 2 [file Data_Sheet_1.PDF]

# MicroRNA profiling of cerebrospinal fluid from dogs with steroid responsive meningitis-arteritis and meningoencephalitis of unknown origin

**Emilio Mármol-Sánchez[1,2†], Pernille Lindholm Heidemann[3†], Hanne Gredal[3*], Susanna Cirera[4*]**

[1]Department of Molecular Biosciences, The Wenner-Gren Institute, Stockholm University, Stockholm, Sweden.

[2]Centre for Paleogenetics, Stockholm University, Stockholm, Sweden.

[3]Department of Veterinary Clinical Sciences, Faculty of Health and Medical Sciences, University of Copenhagen, Denmark.

[4]Department of Veterinary and Animal Sciences, Faculty of Health and Medical Sciences, University of Copenhagen, Denmark.

[†]These authors contributed equally to this work and share first authorship.

***Correspondence:**

Hanne Gredal: hbg@sund.ku.dk

Susanna Cirera: scs@sund.ku.dk

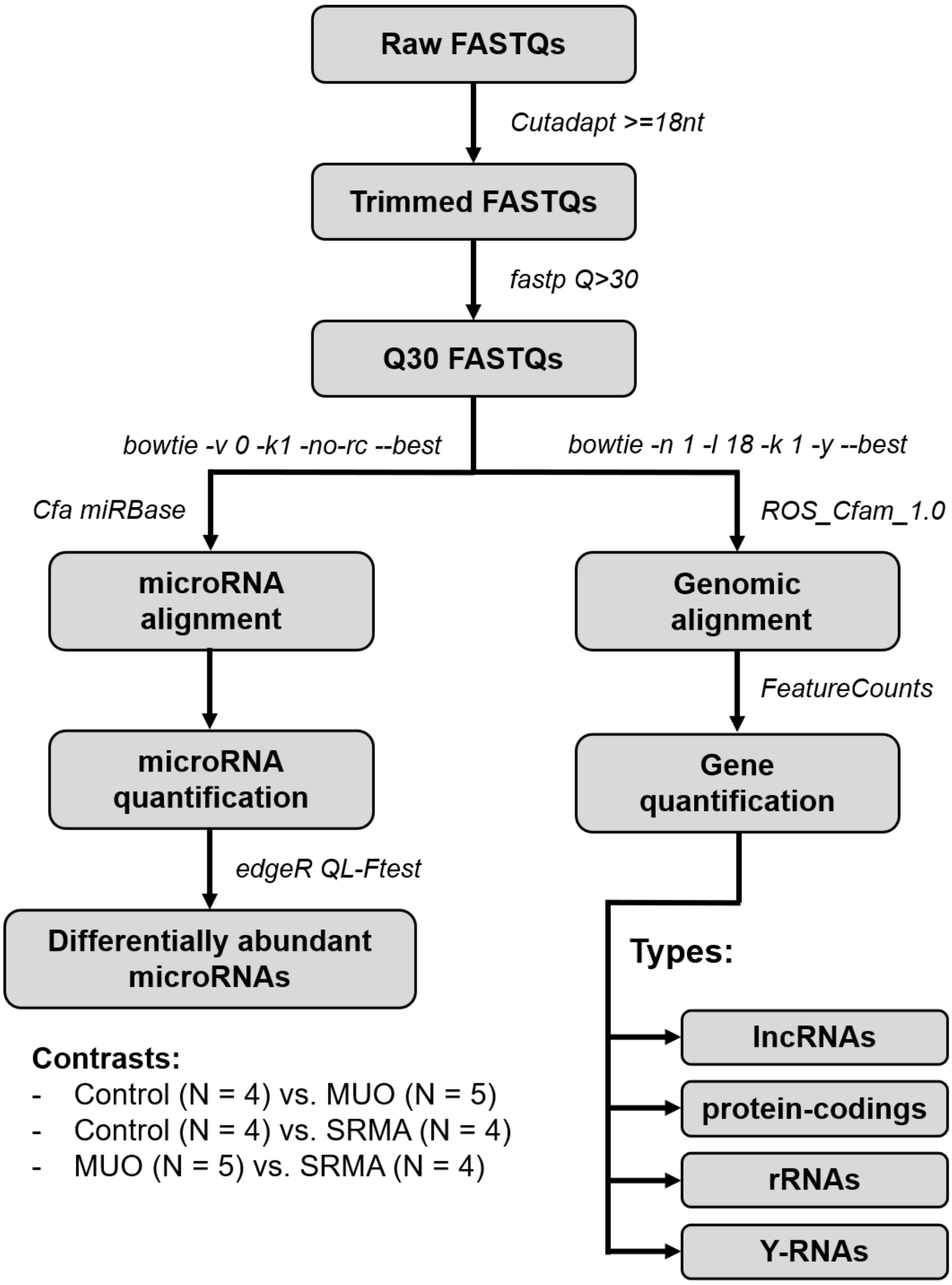


**Figure S1.** Comprehensive pipeline showing the bioinformatics approaches employed for small RNA-seq data pre-processing, mapping, quantification, and differential abundance analyses.

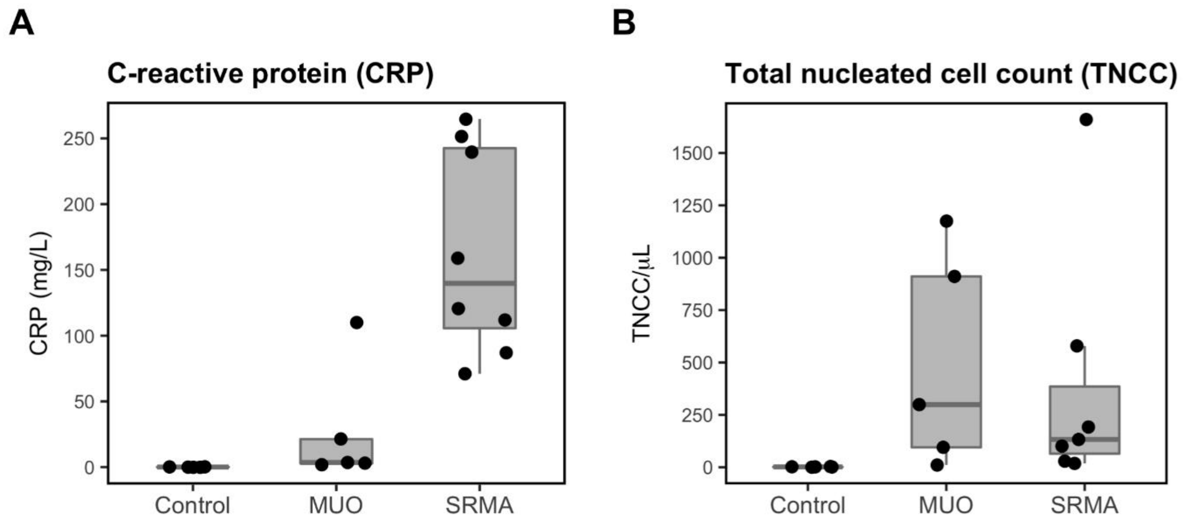


**Figure S2.** Boxplots depicting clinical data for (**A**) C-reactive protein (CRP) and (**B**) total nucleated cell count (TNCC) from control healthy dogs (control, N = 5), dogs with meningoencephalitis of unknown origin (MUO, N = 5) and dogs with steroid responsive meningitis-arteritis (SRMA, N = 8). For the TNCC phenotype, the sample S4 from the SRMA group was considered as an extreme outlier (**Table 1**) and removed for better visualization.

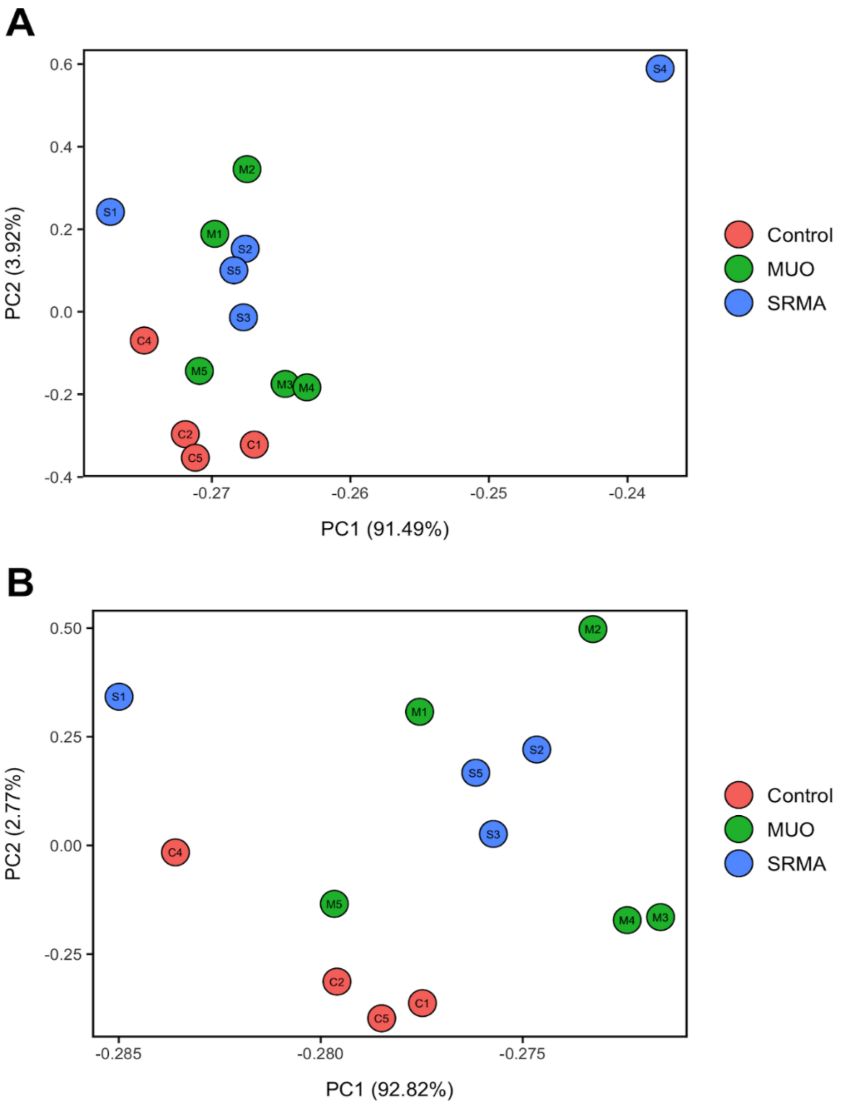


**Figure S3.** Principal component analysis (PCA) of miRNA profiles from cerebrospinal fluid (CSF) samples using small RNA-seq technique. (**A**) PCA of CSF samples based on normalized read counts of miRNA abundance of healthy dogs (Control, N = 4), dogs with MUO (N = 5) and dogs with SRMA (N = 5). (**B**) PCA excluding the S4 sample from the SRMA group, considered as an extreme outlier.
